# The use of hip arthroscopy in the management of the pediatric hip

**DOI:** 10.1093/jhps/hnv070

**Published:** 2015-12-10

**Authors:** Dennis R. Roy

**Affiliations:** 1. Shriners Hospitals for Children, 3101 SW Sam Jackson Park Road, Portland, OR 97239, USA

## Abstract

Arthroscopy of the pediatric hip began in 1977 with a publication by Gross. Interest was relatively slow to develop in the 1980s and 1990s. Coupled with the success of hip arthroscopy in the adult, interest heightened in applying the procedure to a variety of pediatric hip disorders, given that the alternative was an open surgical hip dislocation. The success of this initial group of pediatric hip arthroscopist’s has further expanded the application of hip arthroscopy as the primary or adjunct procedure for the management of intra-articular problems of the pediatric hip.

## INTRODUCTION

Gross [1], in 1977, introduced the era of arthroscopy of the hip in children by utilizing the procedure in 28 patients (24 less than 10-year old) as an adjunct in a variety of pediatric orthopedic conditions including developmental dysplasia of the hip (DDH), Legg–Calve–Perthes disease (LCPD), neuropathic subluxation, prior sepsis and slipped capital femoral epiphysis (SCFE). He utilized a 2.2-mm arthroscope with an anterior portal (lateral to the vessels) and a subadductor portal. Manual traction was used without image intensification. His findings were of interest, but he noted that visualization was limited to the site of capsular puncture and had subsequent difficulty with orientation. Much has changed since his publication. Further experience was published in the 80s and 90s for a variety of conditions (Table I). Interest grew as hip arthroscopy was perceived as a less invasive way to treat intra-articular problems. As more experience was gained, a number of reviews have been published on the use and efficacy of hip arthroscopy in the pediatric age group [6–9], including one publication on patients less than 10 years of age [10].

**Table:** Table I. Historical reports on hip arthroscopy in pediatric disorders

Year	Author	Diagnosis	Number of patients	Age range
1977	Gross [1]	Multiple disorders	27	1–16 + 3 years
1981	Holgersson et al. [2]	Chronic juvenile arthritis	13	12–29 years
1986	Rydholm et al. [3]	Chronic juvenile arthritis	14	7–22 years
1992	Futami et al. [4]	Slipped capital	5	11–13 years
1994	Suzuki et al. [5]	LCPD	19	5–13 years

Most early hip arthroscopy experience in pediatric orthopedics was related to the residual pathology of developmental conditions (DDH, LCP and SCFE) and involved loose body removal and debridement of a torn labrum or ligamentum teres. Pediatric orthopedic surgeons have long dealt with hinge abduction secondary to residual deformity from LCP, SCFE, AVN, etc. [11,12], but a more refined view of the etiology of intra-articular damage was sparked by research published by Leunig and Ganz [13] on the concepts of femoral acetabular impingement (FAI). That knowledge coupled with refinement in arthroscopic techniques, development of specialized instrumentation and improved imaging has lead arthroscopy of the hip to become a standard procedure in adults and an expanded utilization in pediatric orthopedics, given that the alternative is an open surgical dislocation. The technique in the pediatric patient is similar to the adult patient except for the size of the patient and anatomically, for the presence of the proximal femoral, tri-radiate and acetabular growth plates. Pediatric hip disorders have been linked to the development of osteoarthritis of the hip joint, presumably as a consequence of the disease process. Clohisy et al. [14] reported on a cohort of 604 patients, 710 hips with premature, end stage hip disease who underwent primary total hip arthroplasty at or before 50 years of age (average 40 years). Osteoarthritis (47.5%) and osteonecrosis (38.6%) were the two most common diagnoses. Of the osteoarthritis group, 48.4% had DDH, 9.5% had LCP, 6.2% had SCFE and 35.9% were classified as unknown. Of the unknown group; 62.8% had cam morphology, 6% pincer and 30% combined lesions. In the past 16 years, my cases have shifted from primarily LCP, SCFE, DDH and post trauma to at least 60% cam/pincer FAI in the adolescent and young adult population.

## PATIENT ASSESSMENT

The clinical evaluation of the pediatric patient is quite similar to that of the adult patient, especially in patients over age 12 years [15]. There will be a higher percentage of patients who have a pre-existing developmental condition (coxa vara, LCP, SCFE, etc.). The goal is to differentiate intra-articular causes of discomfort from extra-articular etiologies by a thorough history and physical examination. Specific questions regarding location, description of pain, onset, precipitating factors (i.e. flexion for sitting, donning socks and shoes), history of trauma, sports participation especially those involving torsional stress (i.e. martial arts, golf, tennis) and presence of mechanical symptoms should be sought. Remember, pain due to intra-articular pathology is, typically anteriorly, in the groin but may be located posteriorly, laterally, in the thigh or knee due to its referral pattern. Patients may demonstrate the C-sign when describing the location of their pain. Limp may be the only presenting symptom.

On physical examination, note the gait pattern (especially for a Trendelenburg or an antalgic gait) assess the spine for any cutaneous defect or deformity and measure for a potential leg length discrepancy. Check the patient for evidence of ligamentous laxity. The contralateral painless limb should be assessed first. A log-roll maneuver is performed, and pain with that gentle maneuver is suggestive of an intra-articular problem. Record the range of motion and rotational profile of the limb. Obligate external rotation with hip flexion (Drehman’s sign) may be seen with SCFE and FAI. Pain with flexion, adduction and internal rotation (impingement test) is indicative of intra-articular pathology. The Patrick or FABER, McCarthy, dial and posterior impingement tests should be performed. Eliciting snapping of the iliopsoas and iliotibial band should be examined for, as well as tenderness over the greater trochanter. Muscle atrophy and diminished strength should be noted. A good neurologic examination should, likewise, be performed.

## IMAGING

Conventional radiographs should be obtained. A well-centered AP pelvis radiograph is imperative to maximize information as to the bony morphology [16]. The coccyx should be centered on the pelvis and approximately 1.5–3 cm above it. Dysplasia can be assessed as well as acetabular retroversion, coxa profunda and protrusio. A false profile view will provide the degree of anterior acetabular coverage. The frog leg lateral, cross table lateral or Dunn lateral can then be obtained to evaluate the offset of the head neck junction [17,18]. If subluxation is present, reducibility is assessed with an abduction-internal rotation view. The lateral center-edge angle (CEA), anterior CEA, acetabular index (AI), Tönnis or Sourcil angle, the α angle and joint space should be measured.

Advanced level imaging is generally performed to confirm and further delineate the morphology and pathology. Computer tomography is less frequently used due to the concerns of radiation exposure. Magnetic resonance imaging of the hip with or without intra-articular contrast is generally performed. Most centers perform an MR arthrogram of the hip to best assess the labrum and intra-articular structures [19]; however, newer imaging protocols may obviate the need for the intra-articular injection [20]. The articular cartilage can best be evaluated by delayed gadolinium-enhanced magnetic resonance imaging [21].

The indications for arthroscopy of the hip in the pediatric population remain incompletely defined, but its roles as a primary or adjunct procedure continue to expand. Intra-articular pathology amenable to hip arthroscopy includes labral tears, loose bodies and tears of the ligamentum teres, osteochondritis dissecans and intra-articular tumors. The disorders most commonly treated include DDH, LCPD and SCFE, sepsis, epiphyseal dysplasia, post trauma, osteonecrosis, inflammatory arthritis and FAI. The role of arthroscopy in the snapping iliotibial band, iliopsoas or other hip impingements has yet to be defined. The goals of arthroscopy are to identify the pattern and treat the intra-articular pathology as well as correct the morphologic and mechanical abnormality that lead to the intra-articular damage.

## PROCEDURE

**Fig. 1.**
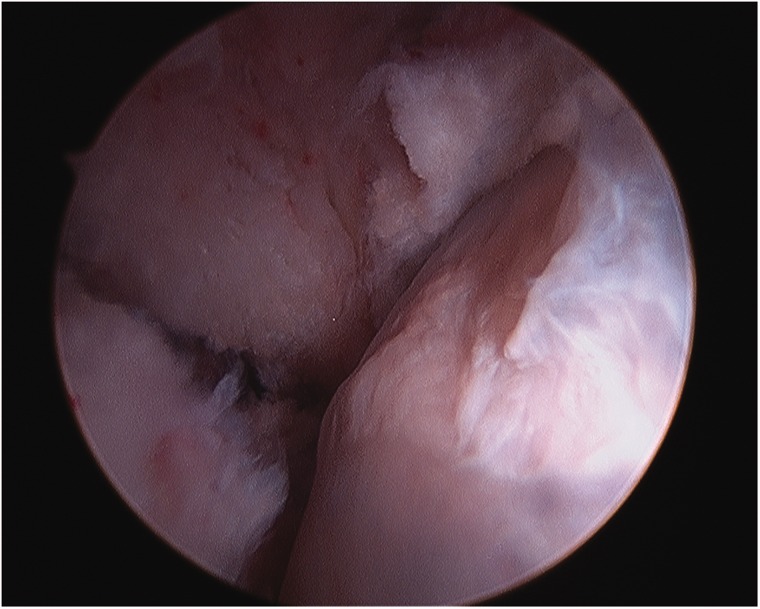
Cam lesion at the head neck junction.

The procedure is performed under general anesthesia with muscle relaxation. Arthroscopy of the hip can be performed in either the supine [22] or lateral decubitus [23] positions. I prefer the supine technique on a standard fracture table. The feet are padded, wrapped and anchored in boots, which often have to be tightened to prevent slippage during the traction. A well-padded perineal post is lateralized against the proximal thigh. Traction is applied to distract the joint approximately 1 cm. Fluoroscopy is utilized to confirm the distraction and to assist in portal placement if necessary. Generally, I use two to three portals, primarily anterolateral and distal mid-anterior with the initial portal being the anterolateral. I distract the joint, then develop my portals, but an alternative is to create the capsulotomy first [24,25], followed by distraction. A complete diagnostic examination is performed after the capsulotomies have been extended with an arthroscopic knife. The 70° arthroscope is used for the majority of the case. Acetabular anatomy has been recently described by Philippon et al. [26]. Central compartment lesions are managed similar to the adult patient, including chondral lesions, labral pathology and cam and pincer pathology. Judicious rim preparation or resection for a pincer lesion is performed to minimize iatrogenic damage to the acetabular ossification centers. Labral lesions are debrided if they are not repairable. Tears of the ligamentum teres are debrided, and the iliopsoas can be tenotomized via the central compartment [27,28] if necessary. Once the central compartment has been managed, the traction is released and the hip flexed. A cam lesion is generally identified by fibrillation and eburnation of the chondral surface in the proximity of the physis (Fig. 1). Resection will need to extend above and below the physis depending upon the maturation of the patient [29]. A dynamic examination is performed to confirm the adequacy of the resection. I generally do not close the capsule. The portals are closed in a standard fashion and a dressing applied. Extravasated fluid will tend to saturate the dressings over the initial post-operative period.

## POST-OPERATIVE MANAGEMENT

The patients are generally admitted as an outpatient surgical procedure with some patients spending the night for pain control. In debridement procedures, protected weight bearing is used until the patient is comfortable and confident. Range of motion is emphasized followed by closed-chain exercises to regain strength. For labral repair and osteoplasty, a continuous passive motion machine is used for approximately 3 weeks, for 8 h a day. Touch down weight bearing with crutches is used for 4–6 weeks and range of motion is encouraged on a stationary bike without resistance for that time period. Strength and endurance are subsequently emphasized. If a micro fracture is performed, the continuous passive motion is used for 6 weeks followed by touch down bearing for another 4–6 weeks. The use of a stationary bike is likewise encouraged followed by strengthening and endurance. Pre-operative signs and symptoms generally improve between 3 and 6 months.

## COMPLICATIONS

Potential complications of hip arthroscopy in the pediatric patient are similar to the adult and include neurapraxia, primarily the lateral femoral cutaneous and pudendal nerves, labral penetration, iatrogenic articular cartilage trauma, implant (suture anchor) -related problems, trochanteric bursitis, hip subluxation, femoral neck fracture, intra-abdominal fluid extravasation and portal would bleeding/hematoma. In the pediatric patient, other potential complications include proximal femoral physeal separation, tri-radiate cartilage injury, growth disturbance (proximal femur, acetabular rim) and osteonecrosis. The overall complication rate is quite similar to that in the adult population [30, 31]. Nwachukwu et al. [32], in a review of 218 pediatric hip arthroscopies, reported a complication rate of 1.8%. This included transient pudendal nerve palsy in two patients, instrument breakage in one patient and a suture abscess in one patient. In addition, I have had transient neurapraxias of the foot related to compression from the traction boots. Nerve dysfunction may be under reported as evidenced by a series reported by Dippman et al. [33] in adults. In their series, 46% of 52 patients reported symptoms of nerve dysfunction in the first week post-operative. At 1 year, 18% had persistent symptoms. The nerve dysfunction was not related to traction time and did not appear to impair patient function or outcome.

In summary, hip arthroscopy in the pediatric population is a safe procedure with a low reported complication rate. Separation of the proximal femoral physis, avascular necrosis or growth disturbance of the hip has not been reported in the pediatric population.

## SPECIFIC DISORDERS

### Developmental dysplasia of the hip

Arthroscopy of the hip has been reported in the management of infant DDH. Gross [1] was able to visualize the pathoanatomy utilizing a subadductor and anterior portals but did not perform any releases or resection to aid reduction in his 4 patients. Hasan and Al-Sabati [34] performed six diagnostic hip arthroscopies in five patients with DDH between ages 1 and 2 years. They were able to visualize successfully through a subadductor portal and reach all structures with instrumentation inserted through a separate portal. Subsequent to these reports, there have been a number of publications (Table II) presenting small series of patients who underwent successful arthroscopically assisted closed reduction [35–38,40]. The author has not utilized hip arthroscopy as an adjunct in the treatment of infantile DDH. There is no intermediate or long-term follow-up as to its efficacy. It may play a role in the reduction of an irreducible hip, in an unstable hip or in a hip that would otherwise need to be immobilized in an unsafe position. Subsequent osteotomy for residual acetabular dysplasia may be required.

**Table:** Table II. Arthroscopic-assisted surgical reduction for DDH

Author	No. hips	Mean age (months)	Follow-up (months)	Notes
Bulut et al. [35]	4	12.8	13.7	Released iliopsoas through small incision
McCarthy and MacEwen [36]	3	14	9	
Eberhardt et al. [37]	5	5.8	13.2	3 hips AVN
Eberhardt et al. [38]	9	21.4	15.4	2 hips AVN
Kitano et al. [39]	10	22.6	64	2 hips AVN70% good evaluation

Hip dysplasia in the teenager and young adult usually presents with the insidious onset of pain. Nunley et al. [40] delineated the presenting symptoms in the young adult. In his series of 57 patients (65 hips), the mean age was 24 years, with a range of 13.4–44.3 years. His criteria for acetabular dysplasia was a CEA < 25°, anterior CEA < 20° and AI >10°. Mechanical symptoms were present in 80%, pain with activity in 88% and night pain in 59%. The pain was located in the groin 72% of the time, lateral thigh 66%, anterior thigh 22% and buttock 18%.

Fujii et al. [41] highlighted the intra-articular findings in symptomatic DDH. In 22 patients, with an average of 16.4 years, he reported 77.8% had cartilage degeneration of the acetabulum and 77.8% had labral tears. Ross et al. [42] reported 63% of central compartment pathology was amenable to arthroscopic treatment. Hips with a CEA < 15° and AI > 20° were associated with more severe chondrolabral pathology.

Hip arthroscopy alone has been used in the management of central compartment pathology in DDH but only with short-term follow-up. McCarthy and Lee [43] reported on 163 patients in 170 hips with an average age of 35 years (12–58 years). Seventy-two percent had labral tears and 59% had cartilage lesions. At 2.5 years post-operative, the mild DDH group (CEA 22-28°) had a conversion rate to arthroplasty of 3%, compared to the cohort with moderate DDH (CEA 16-22°) that had a conversion rate of 54%. Byrd and Jones [44] debrided the torn labrum and reported an improved Harris hip score at 27 months. Domb et al. [45] presented a series in which arthroscopic capsular plication and labral preservation were performed in patients with a CEA of 18-25°. He reported 77% good to excellent results at an average follow-up of 27 months. Parvizi et al. [46], however, published a cautionary note in treating patients with labral tears and DDH with arthroscopy alone. Twenty-five of 34 patients had failure of pain relief, 14 had accelerated arthritis and 13 had migration at follow-up of 3.5 years.

Dorrel and Catterall [47] described the torn labrum associated with hip dysplasia in 1986, stressing that both the labrum and the bony dysplasia needed treatment. Treating labral tears in the pediatric age group with arthroscopy alone if the CEA is less than 20° is not recommended. With a CEA between 21 and 25°, close follow-up for joint deterioration or migration is necessary. Hip arthroscopy in the presence of dysplasia is indicated with mechanical symptoms. I tend to perform the arthroscopy, followed in several weeks by the reconstructive procedure. Concomitant hip arthroscopy and periacetabular osteotomy has been reported [48,49]. Arthroscopy post reconstruction has likewise been reported for intra articular problems as well as femoroacetabular impingement [50].

### Legg–Calve–Perthes disease

**Fig. 2.**
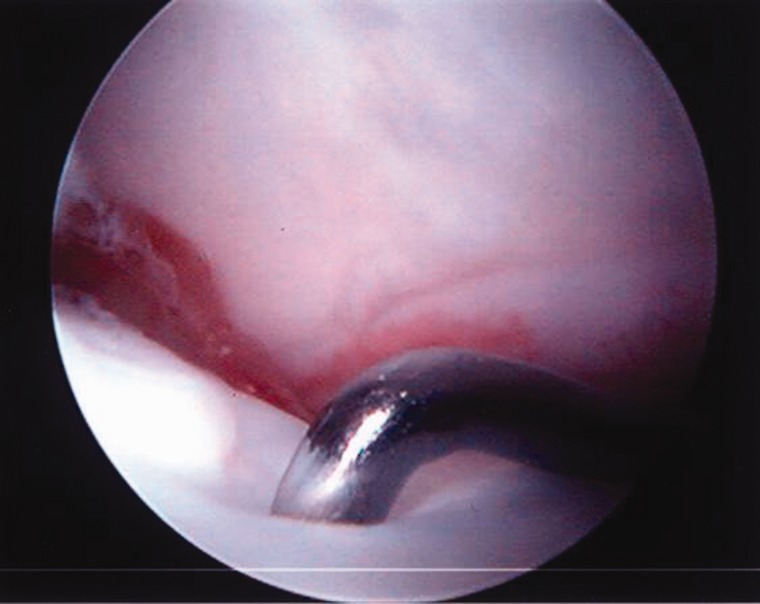
Marked compressibility of the femoral head during the avascular phase of LCPD.

There are a few reports on the arthroscopic findings of the hip in LCPD during the early phases of the disorder. Gross [1] reported areas of flattening of the femoral head, cartilage fibrillation and dilation of the perichandral vascular ring, while Suzuki et al. [5] reported proliferation of the synovium and hypervascularity of the labrum. I have identified marked compressibility of the femoral head (Fig. 2) in a patient who underwent arthroscopy for mechanical symptoms. There appears to be minimal indications for arthroscopy of the hip in patients with LCPD during the unhealed phases of the disorder [51].

Mechanical signs and symptoms may develop due to the sequelae of LCPD in the teen and young adult. The arthroscopist has to potentially contend with a high riding greater trochanter, short neck, large deformed head that may be maldirected due to growth arrest, as well as a potentially abnormal acetabulum. Large capsulotomies with an aggressive capsular resection are usually required for instrument mobility and to visualize the peripheral compartment and for the dynamic examination.

**Fig. 3.**
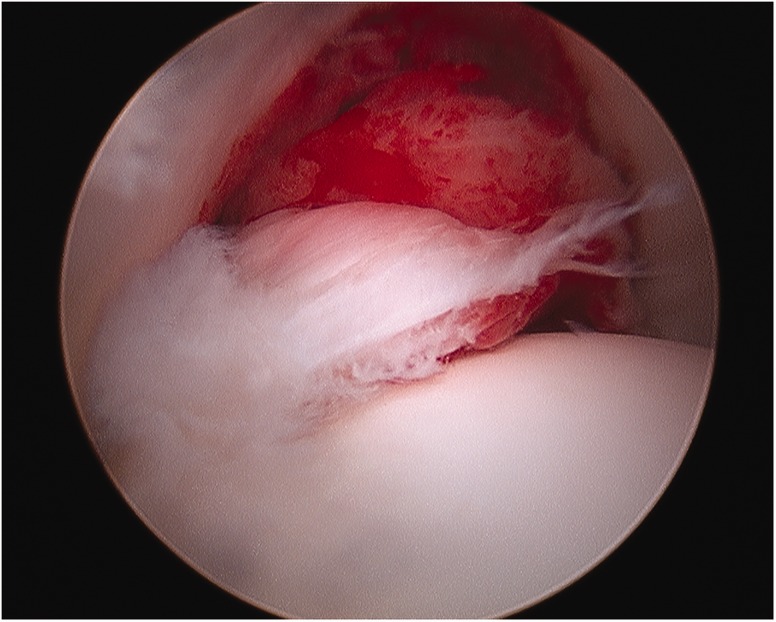
Torn ligamentum teres with foveal synovitis in a patient with LCPD.

**Fig. 4.**
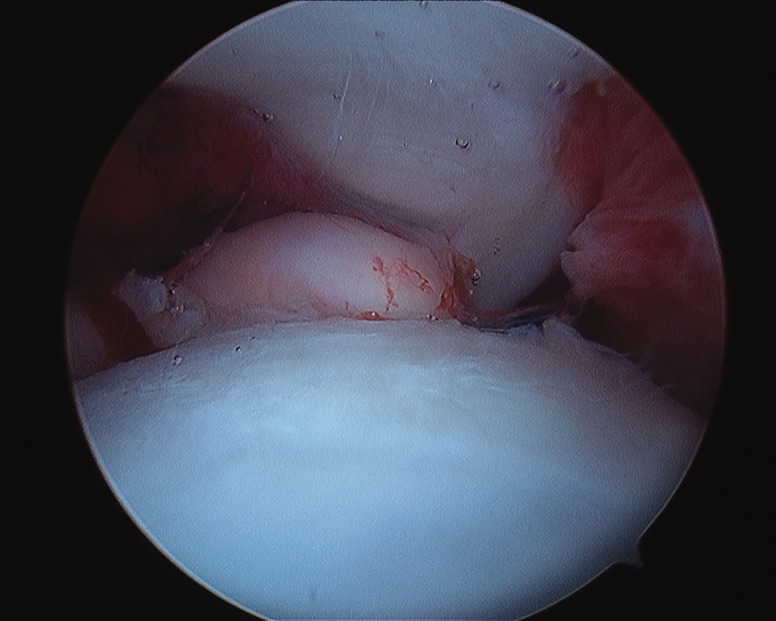
Loose body in a patient with LCPD.

**Fig. 5.**
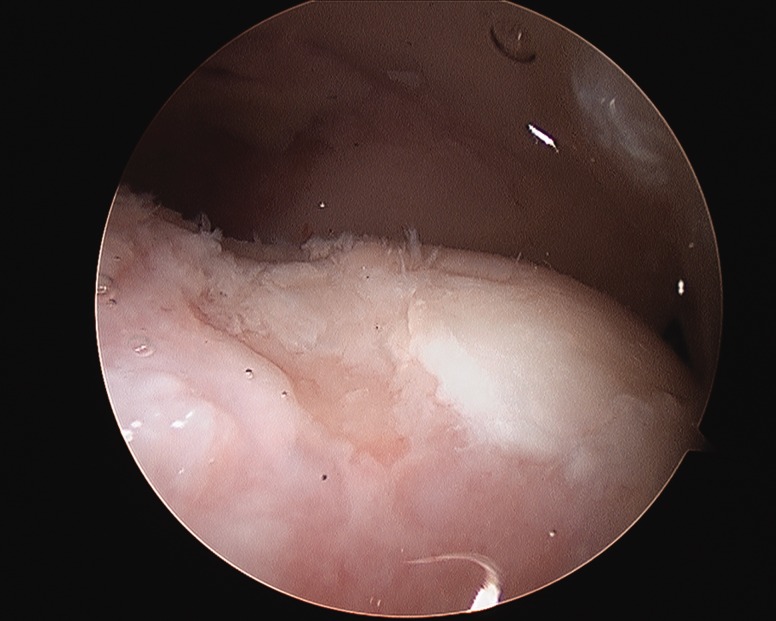
Groove defect of the femoral head in a patient with LCPD.

A plethora of intra-articular findings have been described, including labral tears, tears of the ligamentum teres, osteochondritis dissecans, loose bodies, chondral flaps and groove defects of the femoral head [52,53] (Figs. 3–5). These findings are secondary to the disease process as well as secondary to the conflict between a misshaped femoral head and the acetabulum. Arthroscopy for the management of late sequelae of LCP has been reported [52,54–56] primarily for removal of loose bodies, treatment of chondral flap tears, osteochondritis dissecans and debridement of torn labrum and ligamentum teres. The pattern of damage can assist in planning a subsequent osteotomy or osteoplasty. An arthroscopic osteoplasty of the cam and or pincer lesion is occasionally performed for less severe deformity, otherwise a surgical dislocation and concomitant osteotomy is preferred.

My initial experiences with debridement in the adolescent with LCP were positive. Patients returned to full sporting activities without discomfort, but most had a recurrence of symptoms between 2 and 3 years post procedure necessitating a repeat arthroscopy with or without a proximal femoral osteotomy. Byrd and Jones [44] reported on 23 hips with previous Perthes disease, with an average age of 27 years treated by hip arthroscopy. Modified Harris hip scores improved from 56.7 to 82 at 24-month follow-up.

### Slipped capital femoral epiphysiodesis

Gross [1] reported three hips in two patients with SCFE, two of which had chondrolysis. He suggested that hip arthroscopy with joint irrigation may represent a valuable addition in the treatment of chondrolysis. Futami et al. [4] reported arthroscopic findings prior to pinning of a slip in five patients. He noted synovitis, intra-articular hematoma, erosion of the acetabular cartilage in the anterior superior region and damage to the posterolateral aspect of the acetabular labrum. He also reported cartilaginous erosions and a transverse cleft on the anterior surface of the femoral head. He surmised the hip arthroscopy-aided pain relief.

**Fig. 6.**
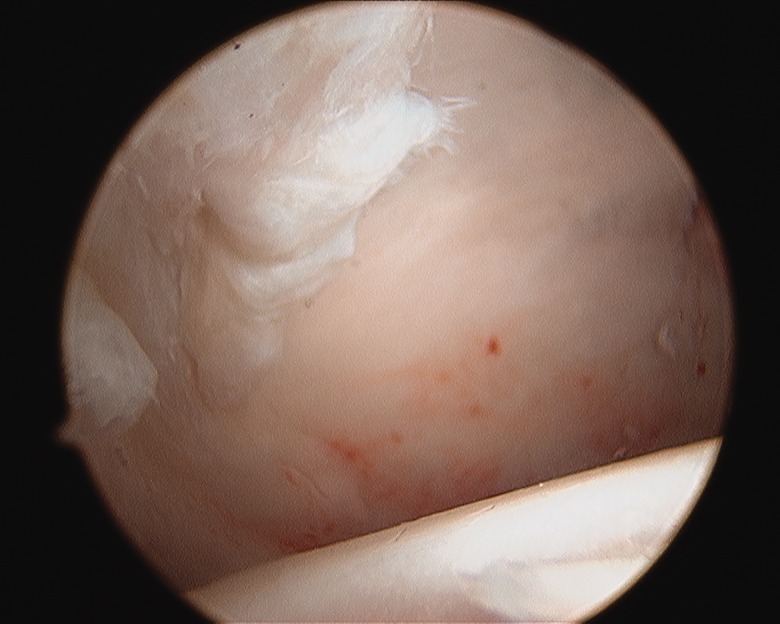
Acetabular cartilage damage in a patient with SCFE.

Treatment of patients with SCFE requires stabilization of the epiphysis and management of the structural deformity. All patients with a post-slip deformity have the potential for FAI. Rab [57] in 1989 utilized a 3D volume/surface computer model to study the geometry of impingement for SCFE and raised the concepts of impaction and inclusion. With impaction, the metaphyseal deformity could lead to erosion of the anterolateral acetabulum. With mechanical levering, there may be erosions in the anterior superior acetabular cartilage and damage or detachment of the posterolateral labrum. His study would suggest more aggressive treatment of the post-SCFE deformity should be considered. Findings identified during open surgical dislocation in SCFE by Leunig et al. [58] and others [59] have revealed a constant pattern of damage to the labrum and either partial or full thickness loss of adjacent acetabular cartilage, confirming the concerns raised by Rab’s [57] studies (Fig. 6).

Not all patients with a post SCFE deformity, however, have symptoms of FAI. In Rab’s computer model, with a posterior slip angle up to 25°, minor impingement could be eliminated with as little as 20° of external rotation. All patients with a SCFE should, however, be viewed as having FAI [60]. Options for the treatment of FAI in SCFE include open reduction with the modified Dunn technique [61,62], flexion interochanteric osteotomy (with or without osteoplasty) [63–65], a combined limited anterior surgical approach with arthroscopy [66] and arthroscopic osteoplasty [67,68]. Chen et al. [68] in a series of 40 hips treated by *in situ* fixation (if previously not stabilized) and arthroscopic femoral neck osteoplasty reported successful results in 88% of patients as measured by relief of pain and obtaining neutral or greater internal rotation measured at 90° of hip flexion. Acetabular morphology needs to be assessed as well for potential acetabular over coverage [69–71].

**Fig. 7.**
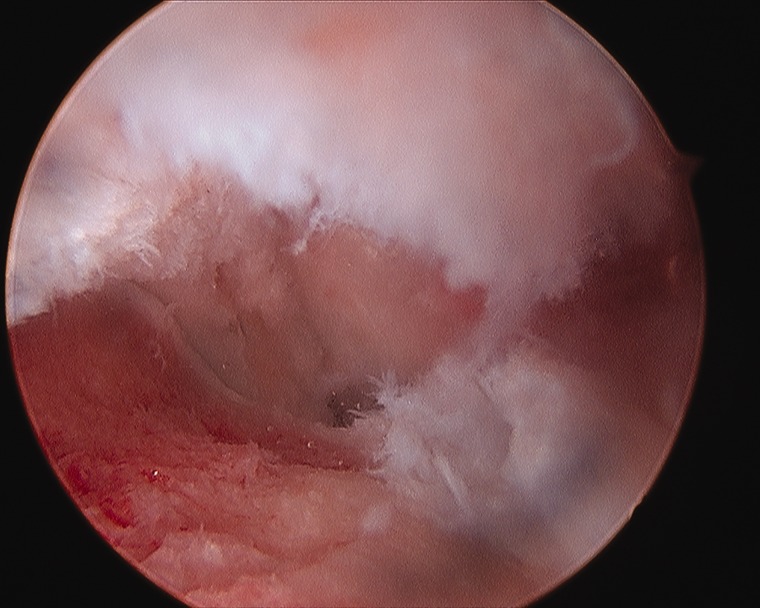
Osteoplasty of the prominent metaphysis in a patient with SCFE.

In summary, arthroscopic osteoplasty with treatment of the intra-articular pathology is appropriate at the time of stabilization of the mild to moderate SCFE (Fig. 7). Limitations to the arthroscopic management relate to the severity of the slip and relative technical difficulties that may be imposed in the obese patient. Impingement may also be caused by the screw used for stabilization [72]. Arthroscopy can be utilized to remove the screw and treat the concomitant hip pathology [73].

### Epiphyseal dysplasia

**Fig. 8.**
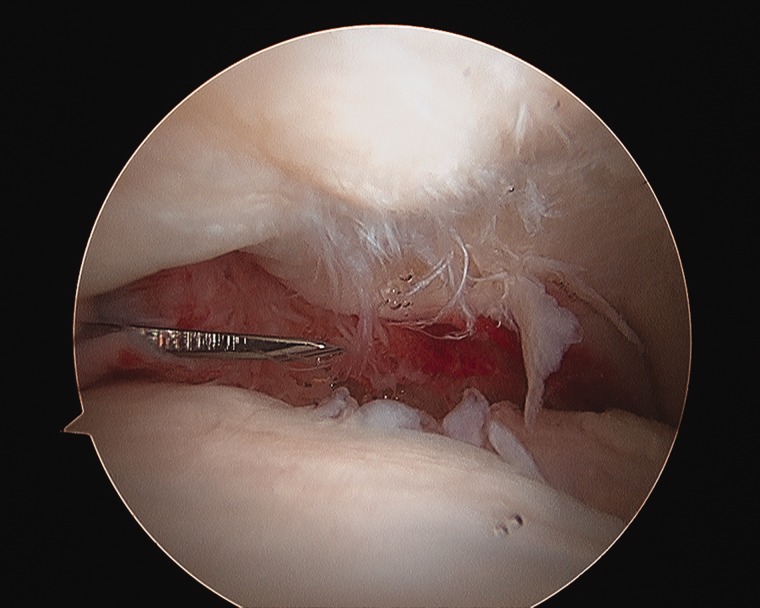
Patient with multiple epiphyseal dysplasia (MED) showing synovitis, labral and chondral damage, loose bodies and femoral head defects.

Multiple epiphyseal dysplasia and spondyloepiphyseal dysplasia are a heterogenous group of disorders caused by a variety of genetic mutations that affects the structure and function of the articular cartilage. Joint pain and deformity tend to be progressive. Mechanical symptoms and signs may develop, causing a change in patient’s baseline symptoms. Loose bodies, labral lesions and chondral lesions have been described [74] (Fig. 8). Chondral avulsion fractures by the ligamentum teres can occur with sudden onset during activities of daily living or pivoting motion. Roy [74] reported on three such patients with a sudden onset of symptoms, including a 3-year-old patient. Arthroscopic treatment allows visualization and management of the pathology with minimal invasiveness. Avascular necrosis has been reported in patients with epiphyseal dysplasia that may further complicate the morphologic pathology and treatment [75].

### Femoral acetabular impingement

FAI is generally divided into three types; cam, pincer and mixed [76]. Cam impingement occurs when a nonspherical portion of an abnormally shaped femoral head or head neck junction conflicts with the acetabulum during motion. In pincer impingement, there is an extension of the acetabular rim, resulting in over coverage of the femoral head. Mixed impingement implies the combination of both cam and pincer impingement.

Sink et al. [77] found the clinical presentation in adolescents as activity-related anterior groin pain. His patients (85% female) exhibited decreased hip flexion and limited internal rotation. All had a positive impingement test. Most patients had mixed impingement (51%), followed by pincer impingement (43%) and cam impingement (6%). Nepple et al. [78] found distinct, sex-dependent disease patterns in patients with symptomatic FAI. Females had more profound symptomatology and milder morphologic abnormalities, while males had a higher activity level, larger morphologic abnormalities, more mixed type FAI morphologies and more extensive intra-articular disease. This would imply that milder morphologic abnormalities should not be over looked in females, and earlier diagnostic methods should be developed, especially in males.

FAI is increasingly being recognized in the teenager and the young adult and has become the most common etiology for my cases. The standard treatment has been an open surgical dislocation with labral repair, rim trimming and osteoplasty of the head neck junction [79].

Philippon et al. [80] has reported on the efficacy for hip arthroscopy in FAI for the pediatric patient and published a preliminary report on 16 patients [81]. In patients with an open physis, he recommended limiting the femoral neck osteoplasty to minimize the risk of an acute SCFE. More recently he reported outcomes of 2–5 years on 60 patients (65 hips) following hip arthroscopy for FAI in the patient aged 11–16 years [82]. He found mixed impingement in 75%. The modified Harris hip score increased from a mean of 57 to a mean of 91, while the median rating for patient satisfaction with their outcome was 10 (range 5–10). He concluded that the arthroscopic management of FAI in the pediatric and adolescent population is a safe procedure with excellent clinical outcomes. Thirteen percent of patients did, however; require a second arthroscopy for capsulolabral adhesions. If impingement morphology is bilateral with only one side symptomatic, I would follow the asymptomatic side at periodic intervals.

### Septic arthritis

Arthroscopic lavage has been successfully utilized in the management of septic arthritis of the hip [83–85] and may be the most common indication for hip arthroscopy in patients younger than 10 years [11]. Metaphyseal drilling can be performed during the procedure if necessary. El-Sayed [85] reported no statistical difference in the outcome of hips treated either by arthroscopic lavage or by open arthrotomy. However, hospital stay was shorter in the arthroscopic cohort.

### Trauma

Arthroscopic removal of retained loose bodies following hip dislocation or fracture dislocation has been reported in the adult [86–89] but has been generally delayed for 3 weeks due to concern for excessive fluid extravasation, either locally or intra-abdominally [89]. In the pediatric age group, the majority of hip dislocations are usually low energy but approximately 20% will have an incongruous reduction [90]. Torn ligamentum teres, osteochondral fragments and the acetabular ephysis with attached labrum have been reported as being interposed, blocking the reduction [91–93]. Open arthrotomy has generally been recommended. Kasiwagi [94] reported on the arthroscopic removal of an avulsion fracture of the ligamentum teres following a traumatic dislocation in a 10 year old.

Roy [8] reported two patients, ages 9 and 16 years, who underwent a delayed arthroscopic treatment following dislocation for a detached posterior labrum in one and the second for torn posterior labrum, torn ligamentum teres and a large impaction lesion on the anterior femoral head. The younger patient became asymptomatic, but the older patient continued with pain after debridement and micro fracture. Arthroscopic management for the incongruous reduction can conceivably be undertaken more acutely, if strict and perhaps limited use of fluid inflow, without a pump is considered.

### Miscellaneous

Hip arthroscopy has been utilized as a diagnostic tool in the staging of avascular necrosis of the femoral head as well as managing any coexisting intra-articular pathologies [95–98]. I have performed hip arthroscopy in patients with mechanical symptoms in conjunction with core decompression. Wells [99] has performed debridement of the osteonecrotic segment under endoscopic visualization in patients with sickle cell disease.

Other reported uses of hip arthroscopy in children and adolescents include excision of benign bone tumors (acetabular fossa exostoses in patients with hereditary multiple exostosis [100] and osteoid osteoma [101]) and inflammatory and synovial based disorders [2,102–104]. I have used hip arthroscopy in two patients who have had a previous Van Ness rotationplasty to treat their intra-articular pathology.

## CONCLUSION

Arthroscopy of the hip has become a safe standard procedure for a variety of conditions in the adolescent and young adult population. The indications continue to evolve and undergo refinement as our knowledge and experience expands. Its use in the very young patient needs further study as to its efficacy. Specialty training in hip arthroscopy is a pre-requisite as there is a learning curve [105]. The types of complications change with the level of experience (e.g. inadequate cam resection) [106]. The anatomy in the typical adult patient tends to be quite uniform, whereas in the pediatric patient, the underlying anatomy is more frequently unique due to the underlying developmental disorders and previous treatment. Outcome measures specific to the pediatric patient needs to be coupled with longer term follow-up, to help refine the indications.

## CONFLICT OF INTEREST STATEMENT

None declared.

